# Real World Data from Catheter Ablation of Ventricular Tachycardias and Premature Ventricular Complexes in a Tertiary Care Center

**DOI:** 10.3390/jcm13082310

**Published:** 2024-04-17

**Authors:** Christian Schlatzer, Jan Berg, Firat Duru, Corinna Brunckhorst, Ardan M. Saguner, Laurent M. Haegeli

**Affiliations:** 1 University Hospital of Zurich, 8091 Zurich, Switzerland; 2 Department of Cardiology, Medical University Department, Kantonsspital Aarau Tellstrasse 25, 5001 Aarau, Switzerland

**Keywords:** ventricular tachycardia, premature ventricular complex, catheter ablation, acute success, clinical outcome

## Abstract

**Background**: Catheter ablation in patients with ventricular arrhythmias (VA), such as ventricular tachycardias (VT) or frequent premature ventricular complexes (PVC), is increasingly considered an effective and safe therapy when performed in experienced centers. This study sought to determine acute success rates and complication rates of ablation procedures for patients with VA in a Swiss tertiary care center. **Methods:** All patients who underwent ablation therapy for VT and PVC at the University Heart Center in Zurich, Switzerland, between March 2012 and April 2017 were included in this analysis. **Results:** A total of 120 patients underwent catheter ablation for VT and PVC (69 and 51, respectively). Seventy percent of patients were male, and the mean age was 55.3 years. The most common indication for ablation was high PVC burden (47.5%), followed by paroxysmal VT (38.3%), ICD shocks (23.3%), incessant VT (12.5%), electrical storm (7.5%), and syncope (3.3%). Acute success rates for VT and PVC ablations were 94.2% and 92.2%, respectively. Rates for complications (including major and minor) for VT and PVC were 10.1% and 7.8%, respectively. Complications occurred only in patients with structural heart disease; no complications were noted in structurally normal hearts. **Conclusions:** Our results suggest that catheter ablation for VT and PVC has high acute success rates with a reasonable risk for complications in the setting of tertiary care centers, comparable to those reported in other studies.

## 1. Introduction

The catheter ablation of ventricular arrhythmias is increasingly considered a treatment option for patients with and without structural heart disease. Symptoms, risk of sudden cardiac death (SCD) and recurrence of ventricular arrhythmias determine the treatment choice. The importance of catheter ablation for the treatment of ventricular tachycardia (VT) and premature ventricular complexes (PVC) increased significantly over the last decades [1,2,3]. Often, catheter ablation is performed as a sole therapy in patients with an idiopathic VT or with idiopathic PVCs [4]. This is different for patients with an underlying structural heart disease, where the implantation of an implantable cardioverter defibrillator (ICD) is the mainstay for SCD prevention [5]. These are life-saving devices; however, they are associated with significant morbidity, including posttraumatic stress disorder [6] or depression [7], due to ICD shocks. Antiarrhythmic drugs are then usually prescribed to reduce the recurrence of VTs and consecutive ICD therapies. However, the efficacy of these drugs is limited, and moreover, significant side effects as well as proarrhythmias may occur [8].

Catheter ablation can be an effective and safe alternative or adjunct to pharmacological therapy to reduce the recurrence of VT in these patients when performed in experienced centers. It is the only treatment option that can change the arrhythmogenic substrate. In recent years, significant progress has been made in identifying and ablating the right targets due to improved techniques and increased experience. 

The purpose of this retrospective analysis of consecutive ablation procedures for VTs and PVCs was to report on acute success rates and procedural complication rates in an experienced tertiary care center in Switzerland. 

## 2. Methods

All patients who underwent ablation therapy for VT or PVC at the University Heart Center in Zurich, Switzerland, between March 2012 and April 2017 were included in this study. 

Prior to ablation in the left ventricle, transthoracic echocardiography (TTE) was performed in all patients to exclude intracardiac thrombi. If feasible, cardiac computed tomography (CT) or cardiac magnetic resonance imaging (MRI) scans were also performed. The data were then merged into the electroanatomic mapping system (CARTO, Biosense Webster, Diamond Bar, CA, USA). 

Acute complete success of VT ablation was defined as abolition of all sustained VTs. Acute partial success was defined by abolition of the clinical VT. At the end of the procedure, the initial induction protocol was repeated to demonstrate non-inducibility of the VT in all patients when the clinical VT was hemodynamically stable. The non-inducibility protocol included 3 extrastimuli and was performed at two different sites of the right ventricle, RV apex and RV outflow tract. In those patients with hemodynamically unstable VT who underwent substrate mapping and substrate-based ablation, success was defined as abolition of all local late potentials and fragmented electrograms in the scar area.

In PVC ablation, complete abolition of the clinical PVC was labeled an acute success. Ability to induce single PVCs at the end of the procedure but to a significantly reduced degree or with a different morphology not resembling the spontaneous clinical PVC was labeled a partial success. 

### 2.1. Catheter Ablation

Anterograde and/or retrograde access routes were chosen at the operator’s discretion, often according to cardiac anatomy and the targeted arrhythmia. Decision for epicardial access could be made by the responsible operator when the substrate was deemed to be epicardial based on etiology of the VT (non-ischemic cardiomyopathies) or in patients after failed endocardial ablations. 

**Figure 1:**
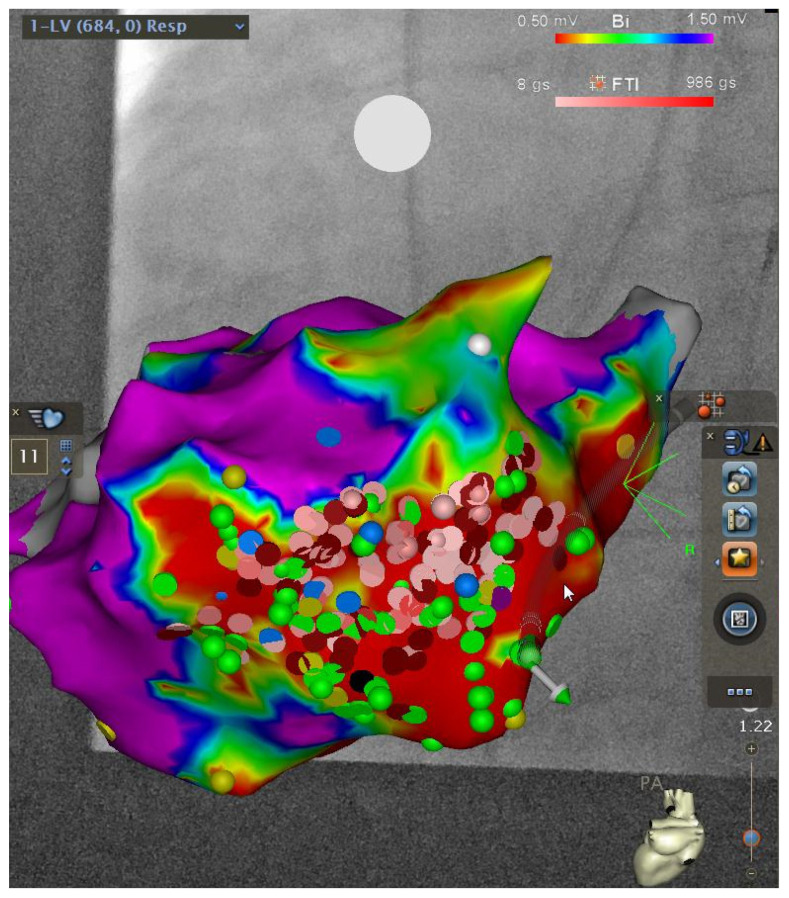
An example of substrate mapping in a patient with hemodynamically unstable VT. A large region of areas with low voltage (red) in the inferior left ventricle is shown. Postero-anterior (PA) view.

**Figure 2:**
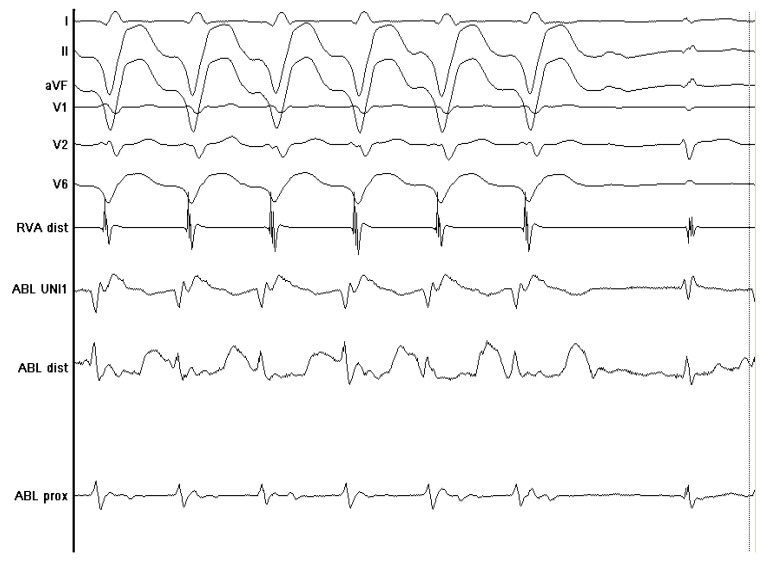
Ventricular tachycardia termination during RF ablation application at the site of late potentials.

Various techniques were used prior to ablation in all patients to map the ventricle for localization of specific targets. In activation mapping, endocardial electrograms were sampled by a mapping catheter and then compared with the timing of the QRS complex on the surface electrocardiogram (ECG). A site that was activated 20 to 40 ms before the onset of the surface QRS complex was considered close to the origin of the VT/PVC. In pace mapping, different sites of the ventricle were stimulated until a QRS pattern was induced that matched the QRS pattern of the spontaneous VT. Pace mapping was used when the relevant VT could not be induced, and a surface ECG of the spontaneous VT was obtained prior to ablation. Entrainment mapping was also used in patients with scar-based VT and involved pacing during a VT at a slightly faster rate than the VT rate. An isolated mid-diastolic potential with a low amplitude from within the critical isthmus of the VT, where entrainment with concealed fusion could be demonstrated, was considered a good site for ablation. Some patients, especially those with structural heart disease, would not have tolerated activation mapping and entrainment due to hemodynamic instability. Inability to induce sustained VT during a procedure was another situation where different techniques came into consideration, categorized as substrate mapping. In substrate mapping, areas of low electrical voltage or delayed potentials were recorded during sinus rhythm (examples shown in Figure 1 and Figure 2). 

### 2.2. Statistical Analysis 

Continuous variables were summarized as mean (SD), and categorical values were summarized as n/total or % of total. Descriptive analyses were performed with Microsoft Excel. To detect differences between groups for acute success and acute complication rates, Fisher’s exact test was performed with GraphPad Prism version 10. A *p*-value < 0.05 was considered statistically significant. 

## 3. Results

### 3.1. Baseline Characteristics

The demographic and clinical baseline characteristics are summarized in Table 1. Patients were predominantly male (84 out of 120 patients; 70%), and the mean age was 55 ± 15 years. Out of 120 patients who were included, 69 (57.5%) were ablated for VT and 51 (42.5%) for PVC. The etiology of arrhythmias was due to structural heart disease in 70 patients (58%), mainly ischemic cardiomyopathy (Table 1). The remaining 50 patients (42%) were diagnosed with idiopathic arrhythmias. 

Indications for ablation treatment are summarized in Table 2. Several indications were possible (33 patients had two indications, and 3 patients had three indications). Antiarrhythmic drugs prior to ablation were used in a majority of patients (86 patients; 72%), mostly patients undergoing VT ablation. Details concerning the use of antiarrhythmic drugs are summarized in Table 1. 

Left ventricular ejection fraction (LVEF) was assessed with TTE in all patients undergoing VT ablation and in 48 out of 51 patients (94%) undergoing PVC ablation. Mean LVEF was 44 percent and 56 percent, respectively. This gap is understandable when different rates of structural vs. idiopathic origins of arrhythmias are being taken into account. 

### 3.2. Catheter Ablation Procedure

In all 120 but one patient, radiofrequency was the energy source used for ablation. In one patient, cryo ablation was used for the ablation of PVC after failure with radiofrequency ablation. Most procedures were performed with local anesthesia or mild deep sedation; however, 26 out of 120 procedures (21%), mostly VT ablations, were performed with general anesthesia. Regarding the entire patient cohort, 66/120 patients (55%) underwent left-sided ablation only. Right-sided only ablation was performed in 43/120 (35.8%) patients. Four patients (3.3%) underwent ablation both in the left and in the right ventricle. In six cases (5%), additional epicardial ablation was performed, and in one patient (8.3%), ablation was performed in the left coronary cusp.

Most of the VT ablations were performed in the LV only (48 out of 69 patients, 69.6%). In 15 out of 69 patients (21.7%), VT ablation was performed in the RV only. In 8 out of 69 patients undergoing VT ablation (11.6% of all VT patients), where an epicardial substrate was expected due to history or ECG morphology, subxiphoidal epicardial access was chosen, and in 6 out of 69 VT patients (8.7%), epicardial ablation was performed (in four cases, ablation was in the LV plus epicardial; in two cases, ablation was in the RV plus epicardial). 

In PVC patients, ablation only in the LV was performed in 18/51 patients (35.3%). In 28 out of 51 patients (54.9%), PVC ablation was performed in the RV only. However, in 4/51 patients (7.8%), PVC ablation was performed both in the left and in the right ventricle. In 1 out of 51 patients (2%), PVC ablation was performed in the left coronary cusp.

In 27 out of 120 procedures (23%) in this study, substrate mapping was the preferred mapping method because of hemodynamic instability during VT. We used a multielectrode mapping catheter (Pentaray, Biosense Webster, Inc., Irvine, CA, USA) from 2015 onward in 31/120 procedures (26%). 

### 3.3. Acute Procedural Success

In the entire patient cohort, ablation procedures were successful in 112 out of 120 patients (93.3%). 

**Figure A1:**
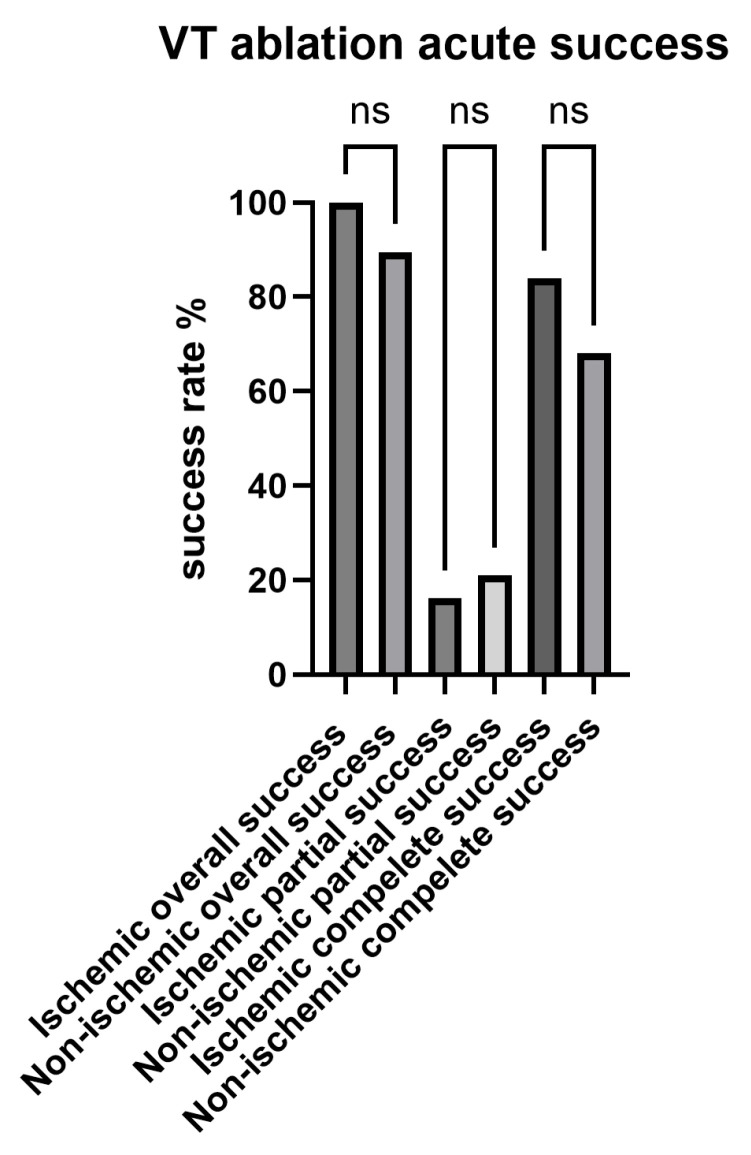
Differences in acute success rates in VT ablation (ischemic vs. non-ischemic); ns = not significant.

In VT ablation, acute success rate was 94.2%. Complete success was achieved in 52 out of 69 patients (75.4%), whereas partial success was achieved in 13 out of 69 patients (18.8%). Success rates in VT ablation were numerically higher for patients with ischemic cardiomyopathy as compared with non-ischemic cardiomyopathy, with complete success in 26 out of 31 patients (83.9%) vs. 26 out of 38 patients (68%) and partial success in 5 out of 31 patients (16.1%) in ischemic vs. 8 out of 38 patients (21.1%). However, the differences in success rates between ischemic and non-ischemic cardiomyopathy were not statistically different (*p* = ns, Appendix A Figure A1). In 24 out of 69 VT ablations (35%), at least one external defibrillation was necessary to treat hemodynamically unstable VT not responding to overstimulation. This was necessary in just one patient (2%) undergoing PVC ablation. 

**Figure A2:**
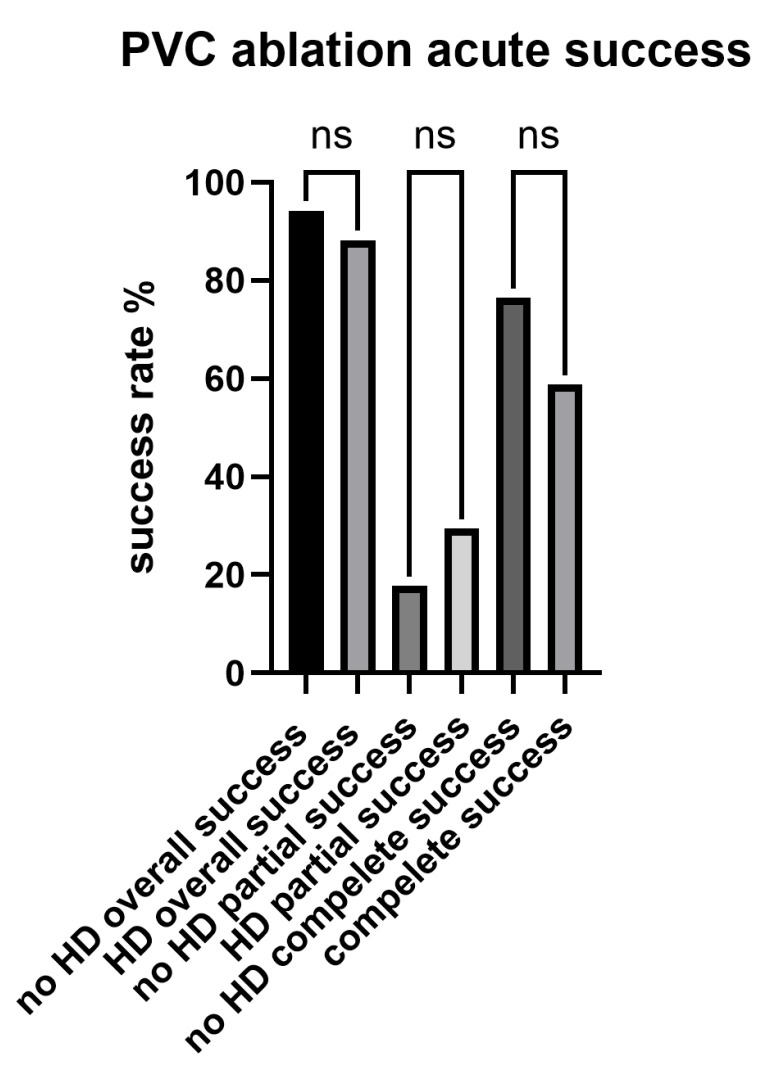
Differences in acute success rates in PVC ablation (no structural heart disease vs. structural heart disease); ns = not significant; HD = heart disease.

In PVC ablation, acute success rate was 92.2%. Complete success was achieved in 36 out of 51 patients (70.6%), whereas partial success was achieved in 11 out of 51 patients (21.6%). Success rates in PVC ablation were numerically higher for patients without structural heart disease as compared with patients with structural heart disease, with complete success in 26 out of 34 patients (76.5%) vs. 10 out of 17 patients (58.8%) and partial success in 6 out of 34 patients (17.7%) vs. 5 out of 17 patients (29.4%). The differences in success rates between normal hearts and structural heart disease in PVC ablation were not statistically different (*p* = ns, Appendix A Figure A2).

In most patients, one single target was ablated. However, in 36 out of 120 procedures (30%), two targets were ablated; in 18 procedures (15%), three targets were ablated and in 7 procedures (6%), four or more targets were ablated. In 32 out of 120 patients (27%), one or more antiarrhythmic drug could be discontinued after successful ablation.

### 3.4. Acute Complications

In 11 out of 120 patients (9%), minor or major procedural complications occurred (Table 3). The most frequent complication was pericardial tamponade, which occurred in 6 patients (5%). Five cases of pericardial tamponade (83%) were resolved by means of percutaneous puncture, whereas one case (17%) had to be resolved by thoracotomy. Vascular complications occurred in two patients (2%). Third-degree AV block occurred in one patient (1%) and left bundle branch block in one patient (1%). In one patient (1%), periprocedural stroke was suspected, as assessed by cerebral MRI. There was no procedural complication leading to death. 

### 3.5. Procedural Safety according to Ablation Site and Approach

In general, there was a trend towards a higher complication rate in patients undergoing left-sided ablation (8 out of 66, 12.1%) when compared with right-sided ablation (1 out of 43, 2.3%). However, this did not reach statistical significance (*p* = 0.085). In patients with ablation in the LV and RV (n = 4) and aortic cusp (n = 1), no complications were reported.

In patients undergoing epicardial ablation (n = 6), the complication rate was 33% (two out of six patients) with one tamponade and one left bundle branch block (epicardial plus endocardial ablation was performed in this case). Thus, we report a numerically higher rate for these patients in comparison to the remaining VT ablation group, where complications occurred in 5 out of 63 patients (7.9%). However, this did not reach statistical significance (*p* = ns).

### 3.6. Procedural Safety according to Arrhythmia Type VT vs. PVC

There were numerically higher procedural complications during VT ablations when compared with PVC ablations (7/69; 10% and 4/51; 8%, respectively). Also, the rate of pericardial tamponade was numerically higher in patients undergoing VT ablations (four patients, 6%) when compared with patients undergoing PVC ablations (two patients, 4%), but both differences were not statistically significant (*p* = ns).

### 3.7. Procedural Safety according to Presence or Absence of Structural Heart Disease

Of note, all 11 complications in our study cohort occurred in patients with structural heart disease in comparison to no complications in patients with the absence of structural heart disease. This difference was statistically significant (*p* = 0.0025).

In the VT subgroup, 76.8% of individuals had structural heart disease, whereas within the PVC group, 33.3% had structural heart disease. Seven complications in VT ablation (100%) occurred in patients with structural heart disease, whereas no complication occurred in patients without structural heart disease, but this difference was statistically not significant (*p* = ns). However, in PVC ablation, where all four complications (100%) occurred in patients with structural heart disease and none in patients without heart disease, the difference was statistically significant (*p* = 0.0095).

## 4. Discussion

### 4.1. Acute Success Rates of Catheter Ablation

Acute procedural success rates were reassuringly higher than 90% both in VT and PVC ablations. Comparing the acute success rates in our study with those of other studies is difficult due to large heterogeneity in patient selection, definition of clinical endpoints, ablation techniques, and other factors. The Cooled RF Ablation System clinical trial was a multicenter, prospective, nonrandomized study, enrolling patients with ischemic cardiomyopathy (ICM) with previous ICD implantation and reported acute success rates of 75 percent [9]. It needs to be noted, however, that acute success was defined as the elimination of all mappable VTs. The Multicenter THERMOCOOL VT Ablation Trial [10] enrolled high-risk patients with hemodynamically unstable and unmappable VTs, thus explaining the lower acute success rates of 49 percent [10]. Another observational study [11] reported an 81 percent rate of acute procedural success in patients with ICM. 

In a study from Germany [12], acute procedural success rates for ablation of PVC were reported to be 67% and 73% in patients <65 and >65 years of age, respectively. The authors of the German Ablation Registry [13] reported acute success rates for PVC ablations of 82% in the overall patient group. In patients without structural heart disease, acute procedural success was significantly higher when compared with patients with structural heart disease (86 vs. 74%, *p* = 0.002). Another study from the United States [14] described acute procedural success rates of 76% in a series of 194 consecutively enrolled patients undergoing catheter ablation for PVC.

### 4.2. Procedural Complications

Procedural complication rates are in part driven by the degree of structural heart disease as well as by comorbidities. In the Multicenter THERMOCOOL VT Ablation Trial [10], high-risk patients with hemodynamically unstable and unmappable VTs were enrolled. This helps explain the reported procedural mortality of 3% in the latter study. At the other end of the spectrum, some authors report no major procedural complications in low-risk patients after the ablation of PVC originating from the right ventricular outflow tract (RVOT) [15].

In our study, two major complications occurred: one pericardial tamponade had to be resolved by means of cardiac surgery, and in one patient, periprocedural stroke was suspected as assessed by cranial MRI. Balancing between interventional success and complications is very important. Our study contains a heterogeneous patient cohort, including patients with structural heart disease (majority of patients undergoing VT ablation) and patients without structural heart disease (majority of patients undergoing PVC ablation). It is important to emphasize that all of the 11 complications in our study occurred in patients with structural heart disease. None of the patients without structural heart disease suffered from complications. We also observed a trend towards a higher number of complications in patients undergoing left-sided ablation in comparison with right-sided ablation; however, the difference did not result in statistical significance. Of note, the complication in our patient cohort that occurred in the group of right-sided ablation was a stroke. But this occurred after retrograde access of the LVOT, and thus, left-sided ablation was finally not performed.

### 4.3. Catheter Ablation vs. Pharmacological Treatment

A multicenter randomized controlled trial (VANISH) [8] compared catheter ablation with escalation of antiarrhythmic drug therapy (AAD) in patients with ICM and implanted ICD who experienced VT despite treatment with AAD. The primary endpoint in the VANISH trial was a composite endpoint of all-cause mortality, appropriate ICD shock after 30 days of treatment or VT storm. There was a 28 percent relative risk reduction, reaching statistical significance, in experiencing the composite endpoint in the 132 patients randomized to the catheter ablation group. Very recently, Della Bella et al. showed in the PARTITA trial that VT ablation after the first ICD shock is associated with a reduced risk of heart failure hospitalization, mortality and recurrent ICD shocks as compared with standard therapy [16]. Arenal et al. found reassuring results in their multicenter randomized SURVIVE-VT trial: patients with ischemic cardiomyopathy and ICD had reduced cardiovascular death, ICD shock, heart failure hospitalization or severe treatment-related complications in comparison to antiarrhythmic drug therapy [17]. These encouraging findings may shift the field more towards catheter ablation in this patient group.

## 5. Conclusions

Our results confirm that catheter ablation for VT and PVC is an effective and safe therapy when performed by experienced professionals in experienced centers. Recognition of this development in the community led to increasing numbers of ablation procedures during the last years, and that will likely further increase. Patients should be considered for catheter ablation for VT at an earlier stage, given that this treatment option can change the arrhythmogenic substrate and, thereby, prevent progression over time. This argument weighs even more in young patients with idiopathic PVC, where a one-time ablation procedure is often considered curative and can prevent patients from taking AAD for long periods and, thus, from significant long-term complications.

In summary, the data in our study suggest that catheter ablation of ventricular arrhythmia in structurally normal hearts is safe, whereas periprocedural complications have to be taken into account when dealing with structural heart disease.

### Limitations

We reported on acute success and complications in the catheter ablation of VT and PVC in 120 consecutive patients over 5 years. However, the population and the results concern very different patients. Moreover, we reported on acute success rates, which can be high after ablation but different a few days after. It is well known that recurrences of VT and PVC are frequent. Mortality after ablation can be delayed. Immediate good results do not mean that the patient will always improve. Our results are limited to acute success since long-term follow-up data are missing in our patient cohort.

## Figures and Tables

**Table 1:** Baseline characteristics.

Baseline Characteristics	All Patients (n = 120)	VT (n = 69)	PVC (n = 51)
Age, years (SD)	55.3 (15)	59 (13.8)	50.3 (15.1)
Male gender, n (%)	84 (70)	57 (82.6)	27 (52.9)
BMI (SD)	27 (4.6)	27.6 (4.8)	26.3 (4.2)
Diabetes mellitus, n (%)	20 (16.7)	17 (24.6)	3 (5.9)
Arterial hypertension, n (%)	50 (41.7)	34 (49.3)	16 (31.4)
Atrial fibrillation, n (%)	13 (10.8)	12 (17.4)	1 (2)
Prior myocardial infarction, n (%)	33 (27.5)	30 (43.5)	3 (5.9)
Prior SCD or ICD shock, n (%)	38 (31.7)	38 (55.1)	0 (0)
Prior VT/PVC ablation, n (%)	20 (16.7)	14 (20.3)	6 (11.8)
Structural cardiomyopathy, n (%)	70 (58.3)	53 (76.8)	17 (33.3)
Idiopathic VT/PVC, n (%)	50 (41.7)	16 (23.2)	34 (66.7)
Acetylsalicylate, n (%)	40 (33.3)	27 (39.1)	13 (25.5)
Oral anticoagulation, n (%)	20 (16.7)	18 (26.1)	2 (3.92)
ACE-I/ARB/ARNI, n (%)	56 (46.7)	42 (60.9)	14 (27.5)
Beta blocker, n (%)	70 (58.3)	55 (79.7)	15 (29.4)
Amiodarone, n (%)	28 (23.3)	27 (39.1)	1 (2)
Mexiletine, n (%)	8 (6.7)	8 (11.6)	0 (0)
Sotalol, n (%)	2 (1.7)	2 (2.9)	0 (0)
Verapamil/Diltiazem, n (%)	11 (9.2)	5 (7.2)	6 (11.8)
Class Ic, n (%)	2 (1.7)	1 (1.4)	1 (2)
ICD/CRT-D, n (%)	47 (39.2)	4 (66.7)	1 (2)

SCD: sudden cardiac death. ICD: implantable cardioverter defibrillator. VT: ventricular tachycardia. PVC: premature ventricular complex. ACE-I: Acetylcholine esterase inhibitor. ARB: Angiotensin receptor blocker. ARNI: Angiotensin receptor neprilysin inhibitor. CRT-D: cardiac resynchronization therapy defibrillator.

**Table 2:** Indication for ablation (multiple indications possible).

Paroxysmal VT	ICD Shocks	Incessant VT	Electrical Storm	High PVC Burden	Syncope
46 (38.33%)	28 (23.33%)	15 (12.5%)	9 (7.5%)	57 (47.5%)	4 (3.33%)

VT: ventricular tachycardia. PVC: premature ventricular complexes. ICD: implantable cardioverter defibrillator.

**Table 3:** Procedural complications, number, and percentages.

Procedural Complications; n (%)	All Patients (n = 120)	VT (n = 69)	PVC (n = 51)
AV block	1 (0.8)	1 (1.4)	0 (0)
Pericardial tamponade	6 (5.0)	4 (5.8)	2 (3.9)
Aneurysma spurium and/or fistula	2 (1.7)	1 (1.4)	1 (2.0)
Stroke	1 (0.8)	0 (0)	1 (2)
Left bundle branch block	1 (0.8)	1 (1.4)	0 (0)
Death	0 (0)	0 (0)	0 (0)

AV: atrio-ventricular. VT: ventricular tachycardia. PVC: premature ventricular complexes.

## Data Availability

The data presented in this study are available on request from the corresponding author.
